# Short-term adaptation during propagation improves the performance of xylose-fermenting *Saccharomyces cerevisiae* in simultaneous saccharification and co-fermentation

**DOI:** 10.1186/s13068-015-0399-4

**Published:** 2015-12-21

**Authors:** Fredrik Nielsen, Elia Tomás-Pejó, Lisbeth Olsson, Ola Wallberg

**Affiliations:** Department of Chemical Engineering, Lund University, P.O. Box 124, 221 00 Lund, Sweden; Department of Biology and Biological Engineering, Industrial Biotechnology, Chalmers University of Technology, SE-412 96 Göteborg, Sweden; Unit of Biotechnological Processes for Energy Production, IMDEA Energy, 28935 Móstoles, Spain

**Keywords:** Yeast, Pre-adaptation, Propagation, Co-fermentation, Lignocellulose, Xylose

## Abstract

**Background:**

Inhibitors that are generated during thermochemical pretreatment and hydrolysis impair the performance of microorganisms during fermentation of lignocellulosic hydrolysates. In omitting costly detoxification steps, the fermentation process relies extensively on the performance of the fermenting microorganism. One attractive option of improving its performance and tolerance to microbial inhibitors is short-term adaptation during propagation. This study determined the influence of short-term adaptation on the performance of recombinant *Saccharomyces cerevisiae* in simultaneous saccharification and co-fermentation (SSCF). The aim was to understand how short-term adaptation with lignocellulosic hydrolysate affects the cell mass yield of propagated yeast and performance in subsequent fermentation steps. The physiology of propagated yeast was examined with regard to viability, vitality, stress responses, and upregulation of relevant genes to identify any links between the beneficial traits that are promoted during adaptation and overall ethanol yields in co-fermentation.

**Results:**

The presence of inhibitors during propagation significantly improved fermentation but lowered cell mass yield during propagation. Xylose utilization of adapted cultures was enhanced by increasing amounts of hydrolysate in the propagation. Ethanol yields improved by over 30 % with inhibitor concentrations that corresponded to ≥2.5 % water-insoluble solids (WIS) load during the propagation compared with the unadapted culture. Adaptation improved cell viability by >10 % and increased vitality by >20 %. Genes that conferred resistance against inhibitors were upregulated with increasing amounts of inhibitors during the propagation, but the adaptive response was not associated with improved ethanol yields in SSCF. The positive effects in SSCF were observed even with adaptation at inhibitor concentrations that corresponded to 2.5 % WIS. Higher amounts of hydrolysate in the propagation feed further improved the fermentation but increased the variability in fermentation outcomes and resulted in up to 20 % loss of cell mass yield.

**Conclusions:**

Short-term adaptation during propagation improves the tolerance of inhibitor-resistant yeast strains to inhibitors in lignocellulosic hydrolysates and improves their ethanol yield in fermentation and xylose-fermenting capacity. A low amount of hydrolysate (corresponding to 2.5 % WIS) is optimal, whereas higher amounts decrease cell mass yield during propagation.

## Background

One of the major hurdles in achieving economical fermentative conversion of lignocellulosic biomass to ethanol is the presence of inhibitory compounds that are generated during thermochemical pretreatment of biomass. Major inhibitors, such as weak organic acids, furaldehydes, and lignin derivatives, have adverse effects on the performance of microbial biocatalysts [1, 2]. Their inhibitory activity affects cellular growth and fermentation behavior, thus decreasing the longevity of the fermenting microorganism, ethanol productivity, and overall ethanol yield of the process [1].

Detoxifying the hydrolysate is one technique of overcoming the limitations that are imposed by such inhibitors [3]. However, many detoxification methods incur additional production costs and add complexity to the fermentation process [4, 5], decreasing the profitability of lignocellulosic ethanol production.

An alternative to detoxification is the use of fermenting microorganisms that can detoxify or tolerate inhibitors in situ without compromising ethanol productivity or yield. A combination of inhibitor-tolerant yeast strains and efficient feed strategies can lower the technological risk in the fermentative step of the lignocellulose-to-ethanol process. Since the economics of fermentation-based bioprocesses depends significantly on the performance of microbial biocatalysts, microbial performance is likely a key to sustainable and cost-competitive production of lignocellulosic ethanol.

Several approaches to developing *Saccharomyces cerevisiae* strains with improved tolerance to inhibitors have been described. Overexpression of homologous or heterologous genes that encode enzymes that confer resistance to specific inhibitors in yeast has improved their tolerance to lignocellulosic hydrolysates [6–8]. Improved tolerance to inhibitors has also been obtained in *S. cerevisiae* strains by evolutionary engineering [9, 10], a method that mimics natural selection by improving cellular properties through iterative genetic diversification and selection. In evolutionary engineering, microorganisms that are subjected to high inhibitor concentrations over extended periods acquire substantial tolerance to inhibitors due to random genetic changes [11].

Pre-emptive exposure to inhibitors can be used during cultivation to provide short-term adaptation and improved performance during fermentation. Whereas changes are incorporated into the genotype of a microorganism in long-term adaptation, short-term adaptation relies on the expressed phenotype and phenotypic heterogeneity. The phenotype that is induced during short-term adaptation primes a microorganism to function in presence of specific environmental factors [12, 13]. Physiologically, adaptation is effected in part by the induction of genes that express a particular resistance phenotype in the presence of sublethal concentrations of inhibitors [7, 8, 14, 15]. The selective pressure exercised by inhibitors during short-term adaptation selects for phenotypes that are more resistant to inhibitors in the substrate.

One method of short-term adaptation of yeast is pre-adaptation—cultivating yeast under conditions that resemble the subsequent fermentation. Pre-adaptation can reduce inhibitory effects and increase the performance of yeast. Several examples of improvements in hexose fermentation have been noted with pre-adaptation of *S. cerevisiae*. Pre-adaptation of *S. cerevisiae* enhances its ability to detoxify or tolerate inhibitors in the media [16]. Yeast that are pre-adapted with hydrolysate liquor during propagation convert hexoses to ethanol faster, and detoxify furfural and 5-hydroxymethyl furfural (HMF) by metabolic conversion considerably faster than yeast that have been propagated in the absence of inhibitors [16]. Short-term adaptation of *S. cerevisiae* with added acetic acid in the pre-culture reduces fermentation times significantly in hexose fermentations with inhibitory levels of acetic acid [17].

In addition, adapting yeast during propagation elicits an adaptive response to inhibitory compounds in the hydrolysate. This is particularly important, because the exact composition of the hydrolysate, especially regarding lignin residues and derivatives, is seldom known and because it is poorly understood which individual compounds are most inhibitory.

Although the impact of short-term adaptation on hexose fermentation has been studied [16–18], the influence on co-fermentation of hexoses and pentoses has not been investigated extensively. Xylose fermentation capacity is affected to a greater extent by inhibitors than hexose fermentation capacity [19]. In using recombinant *S. cerevisiae* with the ability to co-ferment biomass-derived xylose and glucose, the effects of the propagation procedure on xylose and glucose consumption must be considered to realize the desired ethanol. Short-term adaptation during propagation has beneficial effects on the utilization of glucose and xylose in the co-fermentation of bagasse hydrolysates in terms of consumption and conversion [20], suggesting that this method is a feasible approach for increasing the resistance to fermentation inhibitors.

It is an attractive option to combine the use of inhibitor-tolerant strains with short-term adaptation to improve fermentation performance. However, the presence of inhibitors during cultivation on hydrolysate impedes growth [1], resulting in a lower cell mass yield compared to cultivation without inhibitors. Implicitly this means a higher cost of propagation of yeast for a specific fermenter capacity. To improve the economics of the process, the added cost must at least be offset by improved performance of the pre-adapted yeast. Successful pre-adaptation has the potential to decrease yeast loads, shorten fermentation times and increase substrate loads.

This study examined the influence of pre-adaptation on yeast performance and overall ethanol yield from glucose and xylose in simultaneous saccharification and co-fermentation (SSCF) of steam-pretreated wheat straw. The objective was to determine the level of adaptation that is required to promote efficient co-fermentation of glucose and xylose in SSCF while maintaining cell mass yields during propagation. Short-term adaptation was performed by gradually adapting the yeast to inhibitor concentrations that resembled those in the fermentation. The aim was to minimize the hydrolysate requirements in the propagation to preserve high cell mass yields in the propagation step while still acquiring yeast that were adapted to the harsh fermentation environment. Select physiological properties of the cultivated yeast were monitored to identify changes that were induced by the propagation procedure and influenced ethanol productivity and yield during fermentation.

## Results and discussion

In this study we correlated several traits of the propagated cells with their fermentation performance with respect to cell mass yield, cell proliferation, and physiological properties. In the next step, these hallmarks were examined with regard to SSCF to determine their effects on ethanol productivity and yield under relevant process conditions.

### Propagation

Propagation was performed in fed-batch mode after an initial batch culture. During the propagation, hydrolysate amounts that corresponded to 0, 2.5, 5.0, or 10 % water-insoluble solids (WIS) load were added during the late feed phase. Propagation was evaluated in terms of final cell count, cell mass yield, viability, vitality, stress indicators, and expression of genes that conferred resistance to inhibitors.

#### Cell count and cell mass yield

**Fig. 1:**
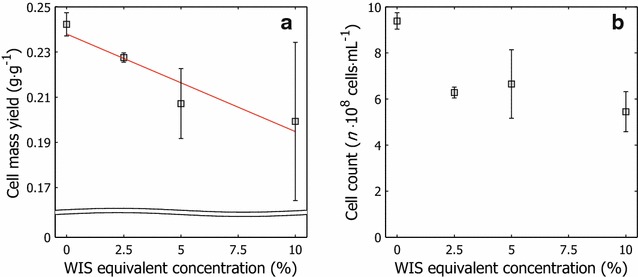
Cell enumeration and cell mass yield at the end of propagation. **a** Cell mass yield after propagation with various amounts of hydrolysate liquor in the adaptation feed solutions, based on supplied fermentable sugars, and **b** hemocytometer-based cell counts after propagation

The cell count at the end of propagation and the cell mass yield in the cultivation step were measured to determine the impact of pre-adaptation on cell proliferation. Cell mass yield declined with increasing amounts of hydrolysate liquor in the feed solutions (Fig. 1a). It decreased by 20 % with inhibitor concentrations in the feed that corresponded to 10 % WIS compared with the molasses reference, which was expected, because cell growth is suppressed by inhibitors that are generated during the thermochemical pretreatment [1]. The molasses solution that was used in the batch cultivation phase contained weak organic acids (1.7 g L^−1^ lactic acid and 0.4 g L^−1^ acetic acid) that inhibit cell growth and potentially act synergistically [21].

The inhibitors from the molasses in the feed solutions (~5.2 g L^−1^ lactic acid and ~1.2 g L^−1^ acetic acid) contributed further to the background inhibitory activity in the propagation medium. The high concentration of inhibitors, especially acetic acid (10.2 g L^−1^) and furfural (7.9 g L^−1^), in the hydrolysate liquor that was introduced to the feed solutions suppressed growth further. Higher inhibitor concentrations in the feed solutions were expected to divert metabolic flux away from growth toward ATP formation to maintain intracellular pH and detoxify the hydrolysate, because weak organic acids lead to intracellular acidification and because cellular detoxification mechanisms are energy demanding.

The presence of xylose in the feed solutions that contained hydrolysate liquor possibly biased the data. Because the employed strain was able to grow aerobically on xylose but preferentially consume hexose sugars, the impact of xylose availability on cell mass yield becomes unclear. However, the xylose-supplemented molasses feed media elicited no significant differences in cell mass yield compared with the molasses reference (data not shown), thus indicating little to no effect of xylose on cell mass yield in propagation.

The differences in cell mass yields were due in part to the cultivation feed strategy. Because the difference in inhibitor concentrations between feed solutions was expected to affect specific growth rates, implementing a fixed feeding strategy for all propagation conditions would have created disparate cultivation conditions, and consequently, certain adapted cultures would have been cultivated under sub-optimal conditions. Overfeeding of substrate, due to a low critical specific growth rate under the prevailing cultivation conditions, causes cells to undergo respiratory–fermentative growth instead of targeted respiratory growth. Thus, cell mass yields will likely decrease as the carbon source is converted aerobically into ethanol—often referred to as the Crabtree effect [22]. Respiratory growth typically leads to a cell mass yield of approximately 0.5 g g^−1^, compared with roughly 0.1 g g^−1^ for aerobic fermentation. Respiratory growth can be ensured through optimization of the propagation feed rate and the use of an exponential feeding profile that keeps the specific sugar addition rate lower than the rate that offsets overflow metabolism.

The final cell counts, shown in Fig. 1b, and cell mass yields had disparate patterns. The cell count for the reference culture on molasses at the end of the cultivation was 9 × 10^8^ cells mL^−1^, and a downward shift to 5.4–6.3 × 10^8^ cells mL^−1^ was obtained with inhibitors in the feed solution (Fig. 1b). By microscopy, larger cells were generated in the presence of inhibitors. Although hemocytometer-based cell counts are prone to experimental error and large spreads, these results indicate lower cell proliferation in the presence of inhibitors.

#### Viability and vitality

**Fig. 2:**
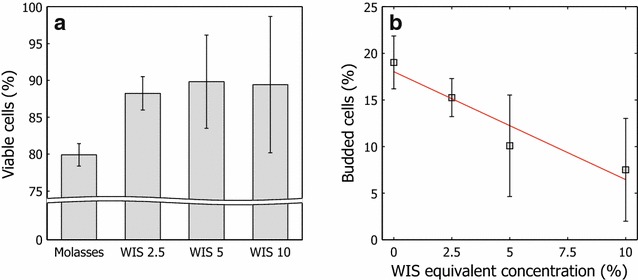
Viability and budding behavior of harvested cells. **a** Percentage of viable cells obtained under various propagation conditions, as determined by methylene blue staining and hemocytometer count. **b** Percentage of budding cells obtained in cultivation with various amounts of hydrolysate liquor in the adaptation feed solutions

Cell viability, the ability of cells to sustain metabolic activity and reproduce, was determined by methylene blue staining and cell counts using a hemocytometer. The percentage of viable cells increased with increasing amounts of hydrolysate liquor in the feed during the fed-batch phase of the cultivation (Fig. 2a). One explanation is that although fewer cells were produced, they were better equipped to survive. However, the frequency of budding cells display an opposing pattern with declining frequency with higher amounts of hydrolysate liquor in the feed (Fig. 2b), likely due to the suppression of cell growth and cell proliferation by inhibitors [1, 14, 19, 23].

It has been suggested that furfural has transient effects and decreases cell replication without inhibiting cell activity [24]. Our results indicate that the metabolic activity improved with short-term adaptation, despite the curtailed ability to reproduce. Further, budding was seen in a small number of stained cells, indicating that some cells were susceptible to the dye but remained viable or that oxygen was present and the dye was reoxidized to its colored state. Both hemocytometer- and methylene blue staining-based counts tend to produce high levels of experimental error [25], hence a variance in the results was expected. Nevertheless, data on viability, although important, are insufficient—cells might be viable but weakly active and are unable to perform in fermentation.

**Fig. 3:**
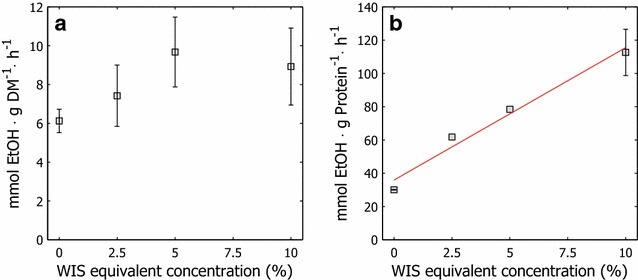
Fermentative capacity of cells propagated with various amounts of hydrolysate liquor. Molar productivity of *S. cerevisiae* KE6-12 propagated with various amounts of hydrolysate liquor in the adaptation feed in relation to **a** inoculated yeast dry weight and **b** yeast protein content

Fermentative capacity tests were performed to assess the vitality of the cultivated yeast. Vitality reflects the physiological state of living cells and, in this instance, refers to the fermentation performance of the yeast. Increased fermentative capacity, in terms of ethanol productivity per gram of yeast dry matter, was obtained with increasing amounts of hydrolysate liquor in the feed (Fig. 3a). The greater fermentative capacity of adapted cells indicates that they were in a more metabolically active state. When the fermentative capacity was expressed as molar ethanol productivity per gram of intracellular protein, this trend became clearer (Fig. 3b). This result indicates that pertinent proteins were synthesized when the cells were subjected to selective pressure. The amount of synthesized intracellular proteins declined with increasing amounts of hydrolysate liquor in the feed solution (data not shown). These results suggest that adaptation enables yeast to produce cells with the proper levels of enzymes and proteins that are needed to maintain high metabolic activity and sufficient energy supplies for energy demanding detoxification and regulation of intracellular pH.

#### Adaptation-induced transcriptional changes

The expression levels of several genes were measured by quantitative PCR (qPCR) in cultures that were adapted with varying amounts of hydrolysate liquor. Genes that conferred resistance to furaldehydes and aliphatic acids and those that promoted growth under toxic conditions (*ZWF1*, *ADH6*, *ALD6*, and *ERG2*) were selected as proxies of adaptation in different cultures.

Previous studies have shown that *S. cerevisiae* converts furfural and HMF into their reduced or oxidized derivatives, which have lower toxicity against *S. cerevisiae* [26]. Cytoplasmic glucose-6-phosphate dehydrogenase, which is encoded by *ZWF1*, and cinnamyl alcohol dehydrogenase, encoded by *ADH6*, converts these furan derivatives into less toxic compounds [7, 8]. Yeast strains that overexpress *ADH6* have also been shown to be able to grow in the presence of toxic aldehyde concentrations [27].

**Fig. 4:**
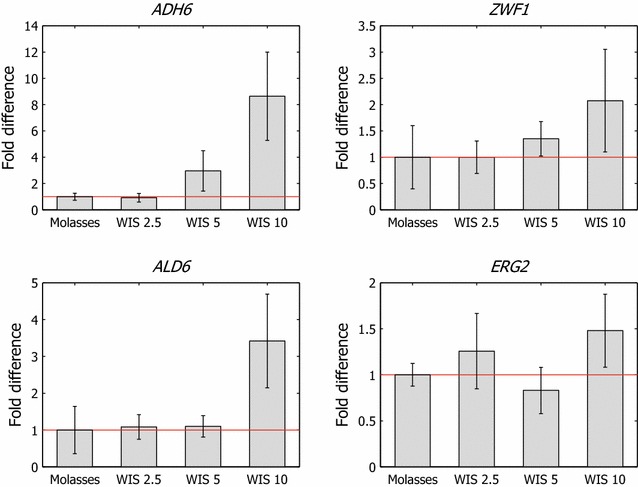
Upregulation of select genes conferring resistance to inhibitors. Relative expression of *ADH6*, *ZWF1*, *ALD6*, and *ERG2* in cell cultures propagated with various amounts of hydrolysate liquor in the adaptation feed solutions

Gene expression of *ZWF1* and *ADH6* was similar between the reference culture and xylose-supplemented cultures (data not shown). Further, the expression of *ZWF1* and *ADH6* did not differ significantly between the reference cell culture and the culture that was pre-adapted with low hydrolysate liquor content (2.5 % WIS equivalent) (Fig. 4a, b). However, *ZWF1* and *ADH6* were upregulated with higher hydrolysate liquor content in the feed (Fig. 4)—i.e., with inhibitor concentrations that corresponded to 5 and 10 % WIS mass fraction.

The cultures generated an adaptive response on a transcription level at furaldehyde concentrations that corresponded to 5 % WIS and were amplified by increased exposure to furaldehydes. *ZWF1* levels were marginally higher with adaptation at inhibitor concentrations that corresponded to 5 % WIS versus the reference state but increased twofold at 10 % WIS. *ADH6* increased threefold at inhibitor concentrations that corresponded to 5 % WIS compared with the reference, which was amplified to an eightfold increase with an inhibitor concentration of 10 % WIS equivalent. The upregulation of these genes reflects the adaptation of cells to environmental factors, which is expected to improve growth and ethanol productivity in the presence of furfural and HMF [14].

The upregulation of *ZWF1* and *ADH6* can affect the distribution of products from the engineered XR/XDH pathway for xylose utilization. Fermentation of xylose to ethanol with recombinant *S. cerevisiae* is slow and exhibits a low ethanol yield, likely due to capacity limitations in the pentose phosphate shunt and an imbalance in redox co-factors created by the xylose catabolism [28]. The redox-neutral process requires NADPH (XR) and NAD^+^ (XDH) [28], which must be regenerated in separate processes. Xylitol formation and excretion can result from an imbalance in co-factors between the NAD(P)H-consuming XR and NADH-producing XDH reactions [29]. Increased activity of NAD(P)H-dependent alcohol dehydrogenase 6 (encoded by *ADH6*) and NAD(P)^+^-dependent glucose 6-phosphate dehydrogenase (encoded by *ZWF1*) in the presence of inhibitors changes the intracellular pool of NAD(P)H [30, 31]. Alterations in the NAD(P)H-pool and the co-factor balance between NAD(P)H and NAD^+^ can influence the product distribution from the engineered XR/XDH pathway and thus the extent of xylitol formation and excretion in recombinant *S. cerevisiae* [30–32].

Aldehyde dehydrogenases, such as the protein that is encoded by *ALD6,* constitute another class of enzymes that have beneficial effects on cell tolerance. The acetaldehyde dehydrogenase that is encoded by *ALD6* plays a critical role in the conversion of acetaldehyde to acetyl-CoA during growth on non-fermentable carbon sources [33] and in the breakdown of toxic aldehydes [14]. It has been shown that the *ALD6*-encoded NAD(P)^+^-dependent aldehyde dehydrogenase is upregulated in the presence of HMF and furfural [34]. In contrast to *ZWF1* and *ADH6*, *ALD6* was upregulated (by threefold) only at inhibitor concentrations that corresponded to 10 % WIS (Fig. 4), which might be an adaptive response to the stress imposed by critical levels of toxic compounds or metabolic readjustment to cope with environmental factors. Park et al. [14] proposed that the overexpression of *ALD6* mediates the recovery of yeast cell metabolism from HMF and furfural inhibition and thus increases ethanol production from lignocellulosic biomass that contained furan-derived inhibitors. Moreover, it has been shown that upregulation of *ALD6* enhances cell growth in media that contains furfural and HMF [14].

*ERG2* mediates the biosynthesis of ergosterol and is one of several genes that are involved in the biosynthesis of plasma membrane lipids that protect against acetic acid [35]. Upregulation of *ERG2* serves as a proxy for changes in the concentration of structural membrane components that confer resistance to acetic acid, for example. There was no significant change in *ERG2* levels at moderate concentrations of inhibitors in the feed (Fig. 4). However, at inhibitor concentrations that corresponded to 10 % WIS, *ERG2* was upregulated 1.5-fold compared with the reference (Fig. 4). The upregulation indicate alterations in the plasma membrane structure to withstand the hostile environmental conditions, which is likely to affect the tolerance of yeast to acetic acid, as reported for other chemical stresses [36].

#### Stress indicators: glycogen and trehalose

The trehalose and glycogen levels in *S. cerevisiae* are believed to be major determinants of stress resistance. These carbohydrates accumulate when growth conditions deteriorate as a means of adapting to various environmental conditions [37]. Trehalose, in particular, has been attributed a role in stress protection, which is a crucial mechanism in the adaptive response to a variety of physical and chemical stresses (e.g., nutritional limitations, heat, oxidative agents, and ethanol inhibition) in *S. cerevisiae* [38, 39]. The relative levels of glycogen and trehalose can be considered indicators of the stress to which cells have been subjected during cultivation [40] but also function as reserve compounds and protect cell integrity against several stressors [39].

**Fig. 5:**
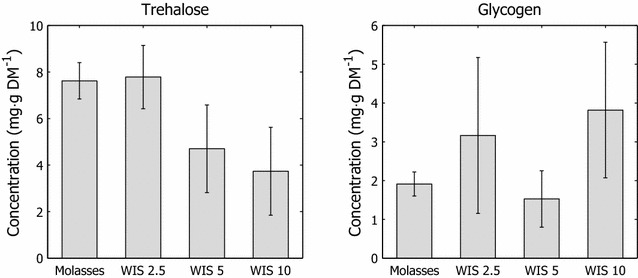
Intracellular trehalose and glycogen concentrations in harvested cells. Intracellular trehalose and glycogen concentrations in cells from propagation with various amounts of hydrolysate liquor in the adaptation feed solutions

As shown in Fig. 5, there were no significant differences in the glycogen content of cells that were adapted with increasing amounts of hydrolysate in the feed. In contrast, intracellular trehalose levels decreased with increasing hydrolysate content during propagation (Fig. 5). Because trehalose is considered to be a stress-induced molecule, the low concentrations of trehalose indicate less stress in adapted cultures due to the inhibitors. Considering the qPCR data, the reduced synthesis of stress-induced molecules might be attributed to an enhanced adaptive response. The decline in synthesized trehalose (Fig. 5) coincides with the upregulation of *ADH6*, *ZWF1*, and *ALD6* (Fig. 4).

**Fig. 6:**
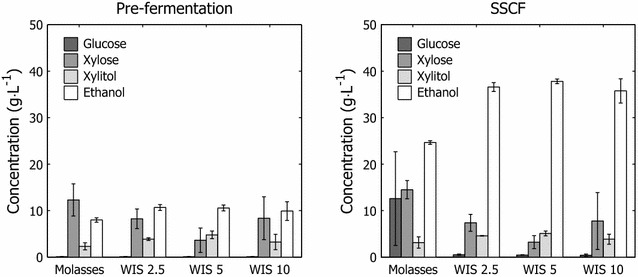
Pre-fermentation and simultaneous saccharification and co-fermentation results. Residual sugars and end-product formation at termination of hydrolysate liquor pre-fermentation at 48 h and SSCF at 120 h

It has been suggested that increased trehalose content in *S. cerevisiae* sustains cell viability during the initial stages of fermentation and thus results in higher carbohydrate utilization rates [41]. Elevated trehalose levels would thus improve the outcomes of the fermentative capacity tests and SSCF evaluation. In this study, this benefit was neither observed in the fermentative capacity tests (Fig. 3) nor in the SSCF experiments (Fig. 6). In these cases, performance improved and trehalose levels declined with increasing amounts of hydrolysate during the short-term adaptation.

### Simultaneous saccharification and co-fermentation

Fermentation performance was evaluated using a hybrid SSCF design, comprising pre-fermentation of the hydrolysate liquor and SSCF with 2 additions of solid material, as described by Nielsen et al. [42]. This design allowed us to study fermentation behavior during hydrolysate fermentation and SSCF under the appropriate conditions for each process and has been applied successfully to obtain high ethanol yields (>90 % of theoretical maximum stoichiometric yield) in highly inhibitory, pretreated lignocellulosic material. Fermentation of steam-pretreated lignocellulosic materials by *S. cerevisiae* KE6-12 has been demonstrated in various fermentation modes [42–45]. In these studies, short-term adaptation was performed during propagation with hydrolysate amounts that resembled the fermentation conditions. However, the effects on fermentation outcomes were not elucidated.

#### Pre-fermentation

**Fig. 7:**
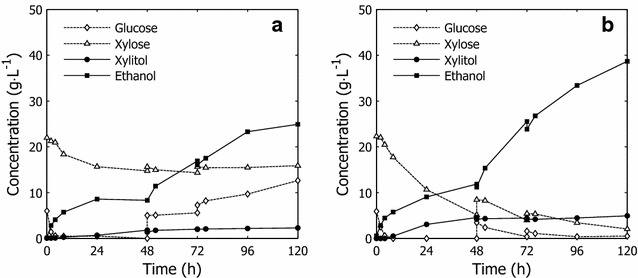
Examples of full cycle fermentation. Full cycle fermentation charts, including pre-fermentation (0–48 h) and SSCF (48–120 h) with cells propagated with **a** only molasses in the feed and **b** molasses and hydrolysate corresponding to 10 % WIS. Both fermentations were performed under the same conditions

All cultures depleted the available glucose during the pre-fermentation (Figs. 6a, 7). The disparity between different cultures appeared in the xylose utilization and end-product formation. Whereas the yeast cultures that were cultivated only on molasses and molasses that were supplemented with xylose utilized 40–50 % of the available xylose (Fig. 6; Table 1), the pre-adapted cultures showed greater xylose utilization and ethanol productivity (Fig. 7). Xylose utilization improved with increasing amounts of hydrolysate liquor in the fed-batch propagation. However, in the pre-fermentations with yeast that was cultivated with an inhibitor concentration that corresponded to 10 % WIS, the variance in xylose utilization increased significantly, correlating with greater variance in cell mass yield, viability, and transcriptional changes with increasing inhibitor concentrations in the feed.

**Table 1:** Pre-fermentation and simultaneous saccharification and co-fermentation results

	Pre-fermentation				SSCF			
	Residual sugars		End-products		Residual sugars		End-products	
	Glucose (g L^−1^)	Xylose (g L^−1^)	Ethanol (g L^−1^)	Xylitol (g L^−1^)	Glucose (g L^−1^)	Xylose (g L^−1^)	Ethanol (g L^−1^)	Xylitol (g L^−1^)
Molasses	BDL	12.3	8.0	2.3	12.6	14.5	24.6	3.1
WIS 2.5 %	BDL	8.2	10.7	3.9	0.5	7.4	36.6	4.6
WIS 5 %	BDL	3.6	10.6	4.8	0.5	3.2	37.8	5.1
WIS 10 %	BDL	8.4	9.9	3.3	0.4	7.8	35.7	3.9

Residual sugars and end-product formation at termination of hydrolysate liquor pre-fermentation at 48 h and SSCF at 120 h

*BDL* below detection limit.

**Fig. 8:**
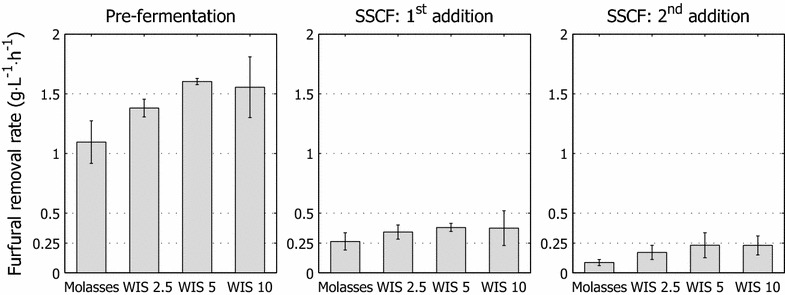
Furfural removal at various fermentation cycle phases. Apparent furfural detoxification rates after discrete addition of inhibitor-containing hydrolysate liquor. The detoxification rate was derived from the average furfural detoxification rate during the first 2 h after the introduction of inhibitors into the bioreactor and represents the removal of furfural from the liquid phase. Values are in relation to 4 g L^−1^ of pitched yeast dry matter

Pre-adapted cultures also produced over 30 % more ethanol than the reference culture, due to improved xylose utilization (Fig. 6; Table 1), which was, however, not mirrored by the ethanol yield. This result is attributed in part to xylitol excretion. The faster utilization of sugar and removal of furaldehydes from the liquid phase by adapted cultures (Fig. 8) demonstrates that the ability of the yeast strain to tolerate and transform inhibitors improved with short-term adaptation.

#### Simultaneous saccharification and co-fermentation

The effect of pre-adaptation became apparent after adding back the lignocellulosic solids during the SSCF. The lower substrate consumption rate in the unadapted cultures caused the accumulation of glucose and xylose in the fermenters, which resulted in low yields due to incomplete substrate utilization (Fig. 6; Table 1). No significant differences in performance were observed between the reference cultures with and without xylose supplement (data not shown), indicating that the presence of xylose in the feed media during propagation had little or no effect on the fermentation during SSCF.

The behavior of the unadapted cultures might be due to lack of adaptation, which would have increased their susceptibility to inhibitors. Based on the high concentration of inhibitory compounds, the longevity of unadapted cultures could be diminished, impairing ethanol productivity. Considering the viability and vitality of the cultures after propagation, the decrease in performance during fermentation can be explained in part by the lower load of viable cells and the lower fermentative capacity in the unadapted cultures. The unadapted cultures were 80 % viable on average compared with 88–90 % for adapted cultures (Fig. 2); further, the molar ethanol productivity in the fermentation capacity test was 6.1 versus 7.4–9.7 mmol g DM^−1^ h^−1^ on average in adapted cells (Fig. 3).

The adapted cultures displayed rapid consumption of glucose and improved xylose utilization (Fig. 7), the latter of which can be linked to some extent to the upregulation of genes that confer resistance to furaldehydes and the ability of, e.g., furfural to act as an electron acceptor in the regeneration of co-factors that are necessary to maintain flux through the engineered XR/XDH pathway [31, 46]. The improvement in fermentation performance was evidenced by the higher ethanol titers and lower marginal xylitol excretion compared with the unadapted reference. Even with pre-adaptation at low inhibitor concentrations, the ethanol conversion improved significantly (Fig. 6 and Table 1)—the reference had an average ethanol concentration of 24.6 versus 35.7–37.8 g L^−1^ for pre-adapted cultures (Fig. 6; Table 1).

The final ethanol concentrations and, thus, ethanol yield increased with pre-adaptation, and no significant difference in ethanol titer was observed between fermentations with cell cultures that were propagated with various amounts of hydrolysate in the fed-batch phase. Increasing amounts of hydrolysate liquor in the feed during pre-adaptation improved xylose utilization, although it was not reflected by higher ethanol titers. Arguably, pre-adaptation had a positive effect on the viability and vitality of the yeast during fermentation, allowing ethanol production to be sustained. This hypothesis is supported by the ability of various cultures to utilize xylose after each addition of solids. Higher rates of xylose utilization were maintained for longer periods with pre-adapted cultures, as were higher apparent furaldehyde detoxification rates (Fig. 8). These measures could be indicators of enhanced viability and vitality of the cells or cells that are better equipped for anaerobic metabolism.

Park et al. [14] reported that transcriptional upregulation of genes that confer resistance to inhibitors correlated with improved fermentative capacity. They found that the highest ethanol productivity was gained with upregulation of *ZWF1* and *ADH6*. On addition of furfural and HMF, *ZWF1* upregulation was associated with the highest specific growth rate and ethanol productivity. Notably, upregulation of *ZWF1*, *ALD6*, and *ADH6* in our experiments occurred during pre-adaptation with inhibitor concentrations that corresponded to 5 and 10 % WIS, but ethanol production was largely unchanged compared with cell cultures that were pre-adapted with 2.5 % WIS equivalent concentration. Because the improvement in fermentation even occurred for cultures that were pre-adapted with low hydrolysate liquor content, there was no correlation between adaptation-induced transcriptional changes and fermentation results. However, it should be noted that the transcription of few genes was investigated, and the resulting phenotypes were the product of a broader range of changes in gene expression.

Nevertheless, the fermentation results in SSCF correlate well with the increase in fermentative capacity and viability of the cultivated yeast at various levels of adaptation. Similar trends were seen in yeast viability at the end of the propagation and in the fermentation results in the SSCF, indicating that the improvement in ethanol yield was due in part to inoculation in the SSCF with higher amounts of viable yeast. Increased viability of the cultivated yeast thus accounted for some of the improvement, whereas the remainder was attributed to improved fermentation performance, as indicated by the increased fermentative capacity. The limiting factor in obtaining high yields was most likely the ability to sustain viability in the culture throughout the fermentation cycle, through extended longevity of the cells or anaerobic growth. The assays did not determine the mechanisms that effected the improvements, but adaptation is clearly beneficial for fermentation in SSCF with steam-pretreated wheat straw.

Another concern is the increased variance in viability with higher hydrolysate liquor content in the feed during cultivation. Although this variability was not fully reflected in the SSCF ethanol titers, it was evidenced by the xylose utilization. Extensive conversion of xylose is a prerequisite for obtaining high ethanol yields in the conversion of lignocellulosic biomass to ethanol—more so when agricultural residues are utilized as substrate. Variability is also an issue from a research and industrial perspective. Reproducible cultivation with low variance ensures consistent performance of the fermenting microorganism and reduces the technological risk. Thus, it would be favorable to adapt the cells with low inhibitor concentrations to minimize hydrolysate consumption and variability in fermentation.

## Conclusions

Adaptation during propagation improves the tolerance of inhibitor-resistant yeast strains and thus increases ethanol yields from glucose and xylose. The improved tolerance of pre-adapted cells resulted in faster and more complete xylose utilization during fermentation. The pre-adapted cells also upregulated genes that conferred inhibitor resistance and experienced greater viability and vitality. The positive effects on ethanol yield in SSCF were observed even for yeast that was adapted at low inhibitor concentrations. Adaptation at higher concentrations of inhibitors than necessary resulted in overall loss of fermentable sugars, due to lower cell mass yield, because more sugars were required to propagate enough yeast for a specific fermenter capacity. Increased variability in cultivation outcome and fermentation was also seen with higher amounts of inhibitors in the pre-adaptation process, which constitutes a technological risk.

## Methods

### Raw material and pretreatment

Wheat straw slurry with a water-insoluble solids (WIS) content of 13.7 % mass fraction was obtained from SEKAB E-Technology AB (Örnsköldsvik, Sweden). The wheat straw was impregnated with dilute H_2_SO_4_ to pH 2 and steam-pretreated at 186 °C for 8 min. The hydrolysate liquor was separated from the solid fraction with a hydraulic press (HP5 M, Fischer Maschinenfabrik GmbH). All solids were retained in the filter cake, and a WIS mass fraction of 48 % was obtained in the solid fraction. The compositions of the solid fraction and hydrolysate liquor are listed in Table 2.

**Table 2:** Composition of hydrolysate liquor and water-insoluble solids

Steam-pretreated material (% of dry matter)	
Glucan	46.8
Xylan	4.7
Galactan	1.7
Arabinan	BDL
Mannan	BDL
Lignin	31.8
Lignin ash	9.5
Hydrolysate liquor (g L^−1^)	
Glucose	11.6
Xylose	36.1
Galactose	3.7
Arabinose	3.8
Mannose	1.5
Formic acid	1.5
Acetic acid	10.2
Levulinic acid	0.05
5-hydroxymethyl furfural (HMF)	1.0
Furfural	7.9

Composition of structural carbohydrates and lignin in the water-insoluble fraction of the pretreated material and sugar composition and prevalence of inhibitory compounds in the hydrolysate liquor. The composition was determined per NREL [53, 54]

*BDL* below detection limit

### Microorganism

The utilized non-commercial *Saccharomyces cerevisiae* KE6-12 strain (Taurus Energy AB) harbors genes from *Scheffersomyces stipitis* that encode xylose reductase (XR) and xylitol dehydrogenase (XDH) and overexpresses endogenous xylulokinase (XK), enabling xylose conversion. The stock culture aliquots contained a mass fraction of 20 % glycerol and were stored at −80 °C.

### Cultivation procedure

#### Pre-cultures

The pre-cultures were cultivated in 250-mL shake flasks with 150 mL of sterile minimal medium that contained 20 g L^−1^ glucose and xylose, 7.5 g L^−1^ (NH_4_)_2_SO_4_, 3.75 g L^−1^ KH_2_PO_4_, and 0.75 g L^−1^ MgSO_4_·7H_2_O. The medium was supplemented with 1 mL L^−1^ vitamin solution and 10 mL L^−1^ trace element solution, the composition of which has been reported by Taherzadeh et al. [47]. The pH of the medium was adjusted to 5.5 with 5 M NaOH solution and inoculated with 300 µL of stock culture aliquots. The pre-culture was incubated at 30 °C on an orbital shaker (Lab-Therm, Kühner) at 180 rpm for 24 h.

#### Propagation

The propagations were performed in 2-L Labfors bioreactors (Infors AG) in a sequential aerobic process: batch cultivation on sugar beet molasses, followed by fed-batch cultivation on wheat straw hydrolysate and sugar beet molasses (Nordic Sugar). The molasses contained 0.411 g g^−1^ of fermentable sugars (sucrose, fructose, and glucose), lactic acid (0.034 g g^−1^), and acetic acid (0.011 g g^−1^). The batch cultivations had a 0.5 L working volume with a 50 g L^−1^ molasses solution that was supplemented with 23.5 g L^−1^ (NH_4_)_2_SO_4_, 3 g L^−1^ KH_2_PO_4_, 2.25 g L^−1^ MgSO_4_·7H_2_O, 33 µg L^−1^ biotin, and 120 ppm Vitahop (BetaTec). The batch cultivation was carried out with a constant aeration rate of 1 vvm and an agitation rate of 700 rpm, and pH was maintained at 5.2. The batch phase was concluded when all sugars were consumed, as indicated by the evolution of carbon dioxide and oxygen in the reactor gas effluent.

Adaptation of the cultivated yeast to fermentation conditions was performed during the fed-batch phase by introducing hydrolysate liquor into the feed solution, as per Alkasrawi et al. [16]. Molasses was the primary carbon source in the feed solutions, and the reference feed solution contained 150 g L^−1^ of molasses. Various amounts of hydrolysate liquor were added to yield inhibitor concentrations in the feed solutions that were equivalent to those in an SSCF with WIS loads of 2.5, 5, and 10 % mass fraction. A constant amount of fermentable sugars (sucrose, fructose, and glucose) was achieved throughout the range of feed solutions by altering the molasses concentration to offset the contribution of hydrolysate-derived glucose. Experiments with reference feed solution that was supplemented with 14.5 g L^−1^d-xylose were performed to determine whether the presence of xylose, without the influence of inhibitors, in the propagation step affected yeast performance.

The feed solution was pulse-added to the bioreactor for 20 h to a final working volume of 1.5 L. The feeding pattern was discretized around a constant dilution rate trajectory (0.056 h^−1^). The agitation rate was maintained at 700 rpm, and the bioreactor was sparged at a constant aeration rate of 1 vvm, based on final volume. The pH was maintained at 5.2 by automatic addition of sterile 2.5 M NaOH solution.

#### Harvest

Samples were withdrawn for various analytical assays and for preparation of inocula for the SSCF experiments. The cultivated cells were harvested by centrifugation (3800×*g*, 10 min) and washed with sterile 9 g L^−1^ NaCl solution. The cell pellets were resuspended in sterile 9 g L^−1^ NaCl solution, yielding a cell dry matter concentration of 120 g L^−1^.

### Hybrid simultaneous saccharification and co-fermentation

The fermentation experiments were performed in sterilized 2-L Labfors bioreactors (Infors AG) with a final working volume of 1.5-L. SSCF was performed per Nielsen et al. [42]. A WIS load of 10 % mass fraction and an enzyme load of 10 FPU g^−1^ WIS^−1^, based on final weight, were applied. The bioreactors were inoculated with a yeast load of 4 g L^−1^ of yeast dry weight, based on the final volume, and the pH was maintained at 5.2 automatically with sterile 2.5 M NaOH solution. The hydrolysate liquor was pre-fermented at 30 °C with an initial addition of 2 FPU g^−1^ WIS^−1^ Cellic CTech2 enzyme solution (Novozymes AS). Half of the solid fraction was added back after 48 h with 8 FPU g^−1^ WIS^−1^ Cellic CTech2 enzyme solution and elevation of the temperature to 35 °C. The remaining solids were added back to the fermenter after 72 h, and the SSCF was terminated after 120 h.

### Analytical procedures

#### Methylene blue staining and cell enumeration

Samples of cultivation broth were dyed with methylene blue (Sigma-Aldrich Chemie Gmbh). The cell suspension was diluted 100 times with 9 g L^−1^ NaCl solution to maintain cell integrity and dyed with 0.3 g L^−1^ methylene blue. The samples were incubated at room temperature for 5 min. Total cells, dyed cells, and budded cells were counted on a hemocytometer in a Bürker chamber.

#### Fermentative capacity

The fermentative capacity test was conducted per Jørgensen et al. [48]. Cells were harvested from 110 mL of broth by centrifugation (3800×*g*, 10 min); washed with 100 mL CBS medium, pH 6.5, without glucose or (NH_4_)_2_SO_4_, [49]; and resuspended in 110 mL of the same media. The cell suspension was transferred to an anaerobic shake flask and incubated at 30 °C on an orbital shaker (Lab-Therm, Kühner). After 5 min, 5 mL of glucose solution (200 g L^−1^) was added. Samples were withdrawn every third minute for 30 min and centrifuged (16,000×*g*, 3 min), and the supernatant was retained, filtered (0.20 μm, GVS Filter Technology Inc), and stored at −20 °C until analysis of the ethanol concentration. The cells in the remaining fermentation broth were harvested to analyze protein content. Ethanol productivity was regressed based on the linear correlation between ethanol concentration and time for the full sample range, and related to the amount of inoculated yeast dry matter and its protein content.

#### Total protein measurements

Cells from the fermentative capacity assay were harvested to analyze protein content by centrifugation (960×*g*, 3 min), washed with sterile distilled water, and frozen immediately in liquid nitrogen. Cell samples were stored at −20 °C until analysis. In preparation for the assay, the cells were thawed, washed twice with distilled water, and suspended in TBS (200 mM Tris, 1.36 M NaCl, pH 7.6) together with acid-washed glass beads. The cells were disrupted in a FastPrep Instrument (MP Biomedicals) for 20 s and kept on ice for 2 min. The cycle was repeated 6 times. The suspension was centrifuged (20,000×*g*, 5 min, 4 °C), and the supernatant analyzed with regard to protein content by Bradford method [50] on a microplate reader (FLUOstar Omega, BMG Labtech). Bovine serum albumin (Sigma-Aldrich, Cat. No. A3803) was used as the standard.

#### Trehalose and glycogen measurements

Cells were harvested from 20 mL of cultivation broth by centrifugation (960×*g*, 3 min), washed twice with 5 mL sterile 9 g L^−1^ NaCl solution, resuspended in 1 mL 20 mM sodium acetate buffer (pH 4.8), and frozen immediately in liquid nitrogen. The samples were then stored at −80 °C until analysis. Approximately 10 mg (dry weight) of cells was resuspended in defined volumes of 0.25 M Na_2_CO_3_ and incubated at 95 °C for 4 h under constant agitation in a thermomixer (Comfort, Eppendorf). Acetic acid (1 M) and sodium acetate (0.2 M) were added to the incubated samples to yield a solution with 62.5 mM Na_2_CO_3_, 0.15 M acetic acid, 0.12 M sodium acetate, and a pH of 5.2.

Aliquots of sample solution were treated with 0.119 U mL^−1^ trehalase (Megazyme K-TREH 11/12) and 2.85 U mL^−1^ of amyloglucosidase (Sigma-Aldrich, Cat. No. A7420). Hydrolysis of trehalose and glycogen was performed under constant agitation overnight at 37 and 57 °C, respectively, in a thermomixer. The supernatant was withdrawn after centrifugation (5000×*g*, 3 min), and the liberated glucose in the trehalose and glycogen assays was measured using the Glucose GOD/PAP kit (Biosis, Cat. No. 000919) with an external glucose standard.

#### Quantitative PCR

Cells from 10 mL of cultivation broth were harvested by centrifugation (960×*g*, 3 min), washed twice with sterile 9 g L^−1^ NaCl solution, frozen immediately in liquid nitrogen, and stored at −80 °C until analysis. RNA was extracted using the RNeasy kit (Qiagen) with DNase treatment per the manufacturer’s protocol. The samples were subjected to reverse transcription and the cDNA was used for qPCR.

Expression of *TAF10*, *ADH6*, *ALD6*, *ZWF1*, and *ERG2* was quantified using Brilliant II SYBRGreen QPCR Master Mix, 0.5 µM of forward and reverse primer, and 2 µL cDNA. The qPCR experiments were performed on a Stratagene Mx3005P. The qPCR program comprised an initial denaturation for 10 min at 95 °C and amplification for 40 cycles of 1 min at 65 °C followed by 1 min at 72 °C for elongation of the amplicons. *TAF10*, used as an internal reference gene to derive ∆*C*_T_ values for the samples, was stably expressed in all samples, because its *C*_T_ value did not vary significantly. The primer sequences were designed from the sequences in the Saccharomyces Genome Database (http://www.yeastgenome.org/) and are listed in Table 3. Data on relative quantification of the genes were evaluated using the comparative ∆∆*C*_T_-method. Fold-differences were expressed as 2^−∆∆*C*T^, where ∆∆*C*_T_ = ∆*C*_T,sample_ − ∆*C*_T,calibrator_.

**Table 3:** Sequences of oligonucleotide primers in the quantitative PCR assay

Gene	Forward primer	Reverse primer
*ADH6*	GTCTTGGTGGTATCGGCAGTATGGGTA	ATGTCGGTAAGGGAGGAAGCACAGACTA
*ALD6*	ACCCAAGAGAAAGAGGCCGTCTACTAAG	GCTCTAAGGTGGTGAAGTTCATGTAGCC
*ERG2*	GCCGAAGTTTACACTCCTGGTATGACTC	TCCCTGGCAGTCAGGTAGACAGTTCTAT
*ZWF1*	GACATTACTGATATCTGCGGGTCTGCT	GGGAACTTGGAAGGGTCTCTGATAAAG
*TAF10*	TACCCGAATTTACAAGAAAAGATAAGA	ATTTCTGAGTAGCAAGTGCTAAAAGTC

#### HPLC analysis

Extracellular metabolites, inhibitors, and sugars were measured by high-performance liquid chromatography (HPLC) on a Shimadzu HPLC system that was equipped with an RID-10A refractive index detector (Shimadzu). Samples for carbohydrate analysis with low pH (from hydrolysates) were pH-adjusted to 5 with CaCO_3_(s) and centrifuged in 10-mL tubes (960×*g*, 5 min). Samples from the fermentation experiments, with adequate pH, were centrifuged in 2-mL Eppendorf tubes at 16,000×*g* for 3 min. All supernatants were filtered through 0.20-μm syringe filters (GVS Filter Technology Inc.) and stored at −20 °C until analysis.

Extracellular metabolites, organic acids, and degradation products in hydrolysate liquors and fermentation broths were analyzed by isocratic ion-exchange chromatography on an Aminex HPX-87H column (Bio-Rad Laboratories) with a Cation-H Bio-Rad micro-guard column (Bio-Rad Laboratories) at 50 °C. The eluent was 5 mM H_2_SO_4_ at a flow rate of 0.5 mL min^−1^. Sugars and xylitol in wheat straw hydrolysate liquor and fermentation broth were quantified by isocratic ion-exchange chromatography on an Aminex HPX-87P column (Bio-Rad Laboratories) with a De-Ashing Bio-Rad micro-guard column (Bio-Rad Laboratories) at 85 °C. Millipore water was used as eluent at a flow rate of 0.5 mL min^−1^.

#### Dry matter and water-insoluble solids content measurements

Water-insoluble solids (WIS) and dry matter content (DM) of solids were measured per standardized laboratory procedures (LAP) that were developed by the National Renewable Energy Laboratory (NREL) [51, 52].

The dry matter mass fraction of the cultivation broths was measured by filtering 10 mL of fermentation broth through a 0.45-μm membrane filter (Whatman Gmbh). The retentate was washed with 15 mL distilled water, and the filters were vacuum-dried for 2 min and dried overnight at 105 °C. Dry samples were cooled in a desiccator for 4 h and weighed on an analytical balance.

#### Composition of hydrolysate liquor and water-insoluble solids

Soluble carbohydrates, monomeric sugars that were released into solution and hydrolysis degradation products were quantified by acid hydrolysis and HPLC per NREL [53]. Further, structural carbohydrate, lignin, and ash contents of the water-insoluble fraction of the wheat straw slurries were measured by two-step hydrolysis method by NREL [54].

## Abbreviations

DM: dry matter
FPU: filter paper unit
HMF: 5-hydroxymethylfurfural
HPLC: high-performance liquid chromatography
LAP: laboratory analytical procedure
qPCR: quantitative polymerase chain reaction
SSCF: simultaneous saccharification and co-fermentation
TBS: tris-buffered saline
WIS: water-insoluble solids
